# Efficacy and safety assessment of two enterococci phages in an *in vitro* biofilm wound model

**DOI:** 10.1038/s41598-019-43115-8

**Published:** 2019-04-30

**Authors:** Luís D. R. Melo, R. Ferreira, Ana R. Costa, H. Oliveira, J. Azeredo

**Affiliations:** CEB – Centre of Biological Engineering, University of Minho, 4710-057 Braga, Portugal

**Keywords:** Bacteriophages, Biofilms

## Abstract

Chronic wounds affect thousands of people worldwide, causing pain and discomfort to patients and represent significant economical burdens to health care systems. The treatment of chronic wounds is very difficult and complex, particularly when wounds are colonized by bacterial biofilms which are highly tolerant to antibiotics. *Enterococcus faecium* and *Enterococcus faecalis* are within the most frequent bacteria present in chronic wounds. Bacteriophages (phages) have been proposed as an efficient and alternative against antibiotic-resistant infections, as those found in chronic wounds. We have isolated and characterized two novel enterococci phages, the siphovirus vB_EfaS-Zip (Zip) and the podovirus vB_EfaP-Max (Max) to be applied during wound treatment. Both phages demonstrated lytic behavior against *E. faecalis* and *E. faecium*. Genome analysis of both phages suggests the absence of genes associated with lysogeny. A phage cocktail containing both phages was tested against biofilms formed in wound simulated conditions at a multiplicity of infection of 1.0 and a 2.5 log CFU.mL^−1^ reduction in the bacterial load after at 3 h of treatment was observed. Phages were also tested in epithelial cells colonized by these bacterial species and a 3 log CFU.mL^−1^ reduction was observed using both phages. The high efficacy of these new isolated phages against multi-species biofilms, their stability at different temperatures and pH ranges, short latent periods and non-cytotoxicity to epithelial cells suggest their therapeutic use to control infectious biofilms present in chronic wounds.

## Introduction

The skin is the largest organ of the human body, is the main protective barrier against the external environment. Injury or illness may cause loss of skin integrity (wound) that in some cases may become chronic. Chronic wounds are defined as wounds in which the normal reparative process failed and the anatomic and functional integrity is not produced within a period of three months, as a consequence of a pathology or of a microbial invasion occurrence in the wound^1,2^. Worldwide, these wounds are responsible for considerable morbidity and significantly contribute for an increase in health care costs. In the USA, chronic wounds affect annually around 5.7 million people (≈2% of population), and their treatment has US$20 billion of annual expenses^3^. Moreover, with an increasing population aging and the lifestyle changes, a rise of new cases is expected in upcoming years^4^.

Pathogenic biofilms are associated with many chronic diseases, including chronic wounds^5^. These infectious biofilms are described to be present in 6% of acute wounds, with the numbers increasing to 90% in chronic wounds^6^. The wound is a favorable environment for biofilm development, and bacteria can stimulate inflammatory response and delay the wound cure^7^. Infectious biofilms are usually of polymicrobial nature, and several studies identified *Enterococcus* sp. as one of the most frequent bacteria present in chronic wounds^8–10^. Indeed, *Enterococcus* sp. is commonly isolated from diabetic foot ulcers, venous leg ulcers and pressure ulcers^11,12^. *Enterococcus faecalis* and *Enterococcus faecium* are Gram-positive facultative anaerobic cocci that form chains of various lengths. They are commensal inhabitants of the gastrointestinal tracts of humans and other mammals with the capability to survive in harsh environments, including hospitals^13^.

Several factors are involved into the conversion of enterococci into a high relevant clinical problem. Besides their intrinsic capacity to resist several antibiotics, including vancomycin, enterococci strains are involved in the dissemination of determinants of antibiotic resistance^13^.

Very recently, the World Health Organization (WHO) published a list of priority pathogens resistant to antibiotics. In the high priority list are strains such as *E. faecium*, resistant to vancomycin^14^. In the same report, WHO encourages the scientific community and pharmaceutical industries to focus on the development of new antimicrobials to combat antibiotic resistant pathogens^14^.

In recent years, the use of bacteriophages (phages) has re-gained interest mainly due to their host specificity and bacteriolytic activity against antibiotic-resistant strains and biofilms^15^. Several studies report the success of natural or genetically modified phages against enterococci biofilms^16–18^.

In the present study, two newly isolated *Enterococcus* sp. phages were combined to target dual species biofilms formed in wound simulated conditions. Additionally, the effect of phages in epithelial cells was assessed.

## Results and Discussion

### Max and Zip are two newly isolated enterococci phages

Phages are ubiquitous in nature and they can be found wherever their host is present, therefore it is expected to isolate them in almost every environment^19^. In this work, phage vB_EfaS-Zip (Zip) infecting *E. faecium* and vB_EfaP-Max (Max) infecting *E. faecalis* were isolated using sewage water from wastewater treatments plants ETAR Braga (Frossos).

**Figure 1:**
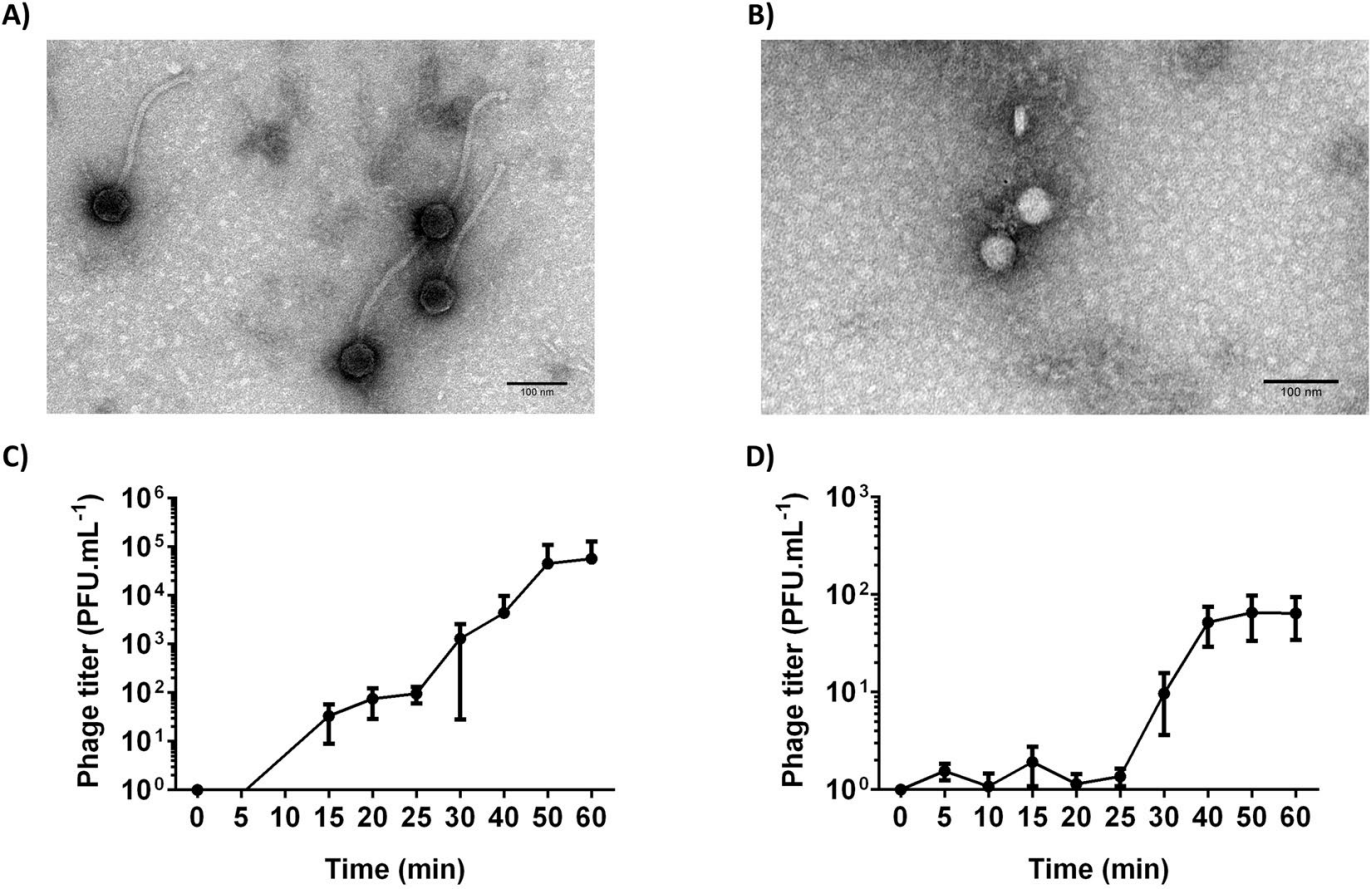
Morphological features and one-step growth curve of enterococci phages Max and Zip. Transmission electron microscopy micrographs with magnification: x150,000 showing purified phage particles of (**A**) *E. faecalis* phage Max; (**B**) *E. faecium* phage Zip. Phages were negatively stained with 2% (*w*/*v*) uranyl acetate. Scale bar represents 100 nm. One-step growth curve parameters of (**C**) phage Max with *E. faecalis* strain Efa1; (**D**) phage Zip with *E. faecium* strain C410. Error bars represent standard deviations from three independent experiments performed in duplicate.

To better characterize the phages, their morphologies were observed by TEM and both revealed to belong to the *Caudovirales* order. According to the morphological evaluation^20^, *E. faecalis* phage Max belongs to the *Siphoviridae* family, having a non-contractile tail with 220 nm in length and 12 nm in width and a capsid with 58 nm in diameter (Fig. 1A). *E. faecium* phage Zip has a capsid with 46 nm in diameter and a short, non-contractile tail with 19 nm in length, and consequently belongs to the *Podoviridae* family (Fig. 1B).

To further characterize both phages’ infection cycles, one-step growth curves were performed. Phage Max showed a latent period of 10 min with an average burst size of 38 PFU per infected cell (Fig. 1C). Although the burst size is smaller than other siphoviruses such as IME-EF1 (60 PFU per infected bacteria), the latent period of Max is shorter than the referred phage^21^. Phage Zip has a latent period of 25 min with average burst size of 52 PFU per infected cell (Fig. 1D). A morphological similar *E. faecium* phage, vB_EfaP_IME199, had a similar latent period, but a smaller burst size^22^.

It has been described that phages with short latent periods have more efficient replication cycles which are related with the success of *in vitro* studies^23^. Podoviruses are usually associated with short latent periods are higher burst sizes than more complex phages, such as myoviruses^23^.

### Enterococci phages have a wide host lytic range among clinical isolates

The lytic spectra of both isolated phages was tested against a panel of enterococci strains to study their host range (Table 1). Even with different bacterial species as hosts, the host range of both phages is quite similar. *E. faecalis* phage Max can lyse 12 out of the 16 *E. faecalis* (75%) strains tested, and also 2 of the 13 *E. faecium* strains tested (15%). *E. faecium* phage Zip has a narrow host range in *E. faecium* lysing 23% (3 out of 13) and 69% of the *E. faecalis* strains tested (11 out of 16). Moreover, both phages did not show any lytic effect on the other Gram-positive species tested.

**Table 1:** Lytic spectra of enterococci phages vB_EfaP-Max (Max) and vB_EfaS-Zip (Zip) against a panel of clinical isolates.

Species	Strain	Source	Max	Zip
*Enterococcus faecalis*	CECT 184	Milk	+	+
	V583	Blood culture	−	+
	LMV-034	Urine	−	−
	LMV-035	Peritoneal liquid	+	+
	LMV-036	Urine	+	+
	LMV-038	Urine	+	+
	LMV-039	Urine	+	−
	LMV-040	Urine	+	+
	LMV-056	Urine	−	−
	Efa1	Urine	+	+
	Efa2	Blood culture	+	−
	Efa3	Urine	−	+
	Efa4	Blood culture	+	+
	Efa5	Pus	+	+
	Efa6	Pus	+	−
	Efa7	Urine	+	+
*Enterococcus faecium*	C410	Unknown	−	+
	LMV-037	Urine	−	−
	LMV-041	Unknown	−	−
	LMV-042	Perineal exudate	−	−
	Efe1	Blood culture	−	−
	Efe2	Blood culture	+	+
	Efe3	Perineal exudate	+	+
	Efe4	Urine	−	−
	Efe5	Pus	−	−
	Efe6	Urine	−	−
	Efe7	Skin exudate	−	−
	Efe8	Pus	−	−
	Efe9	Urine	−	−
*Enterococcus gallinarum*	Ega1	Pus	−	−
*Staphylococcus aureus*	ATCC 25923	Clinical isolate	−	−
	CECT 239	Human lesion	−	−
*Staphylococcus epidermidis*	RP62A	Catheter-associated sepsis	−	−
*Listeria monocytogenes*	CECT 5725	Chicken	−	−

Phage Max has shown a wide host range among *E. faecalis* isolates. Furthermore, the lytic effect demonstrated in *E. faecium* was already reported on the siphovirus IME-EF1^21^. The narrow host range for *E. faecium* phages was already reported for phage IME-EFm5^24^. Nevertheless, the fact that Zip is a phage active against *E. faecalis* is quite relevant, as a similar phage, IME-199, is reported as infecting specifically *E. faecium*^22^. Casey *et al*., discussed recently that the infective success of different phage morphologies seems to be host specific^23^. Therefore, there are no biological markers that could be used to predict the therapeutic success of a phage.

### Max and Zip are stable in wounds pH and temperatures

Physico-chemical factors, such as temperature and pH have been described to influence phage survival and persistence^25^. To assess the potential of both enterococci phages for therapy, stability tests were made exposing phages to different temperatures and pHs for 24 h to simulate putative product development steps and storage conditions.

**Figure 2:**
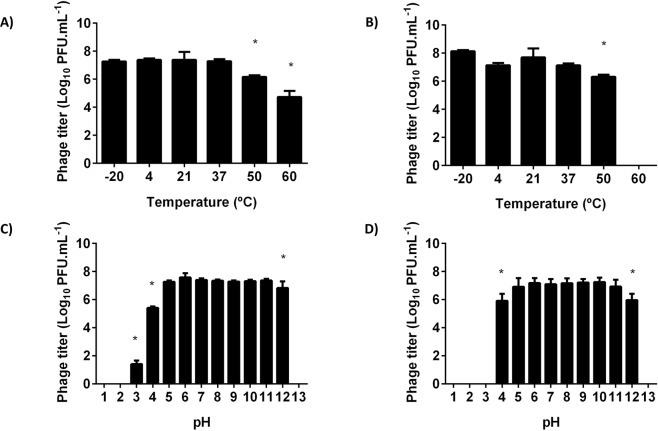
Thermal and pH stability test of enterococci phages Max and Zip. (**A**) Thermal stability of *E. faecalis* phage Max; (**B**) Thermal stability of *E. faecium* phage Zip; (**C**) pH stability of *E. faecalis* phage Max; (**D**) pH stability of *E. faecium* phage Zip. Temperature experiments were performed for 24 h at pH 7. pH experiments were performed for 24 h at room temperature (21 °C). Error bars represent standard deviations from three independent experiments performed in duplicate. *Statistically significant (p < 0.05) conditions between control (4 °C or pH 7) and test assays.

Temperature plays a fundamental role in phage attachment, genetic material ejection and phage multiplication^26^. A thermal stability test was carried out to determine the heat resistance of isolated phages at pH 7.0. Both phages were stable after 24 h, at −20 °C, 4 °C (control), 21 °C and 37 °C, showing a concentration of about 7 log CFU.mL^−1^ (Fig. 2A,B). At 50 °C, both phages lost their titer in about 1 log CFU.mL^−1^ (p < 0.05). At 60 °C *E. faecalis* phage Max concentration decreased about 2 log CFU.mL^−1^ (p < 0.05) (Fig. 2A), while phage Zip was completely inactivated at (Fig. 2B). The results are in agreement with those reported by Lee *et al*.^27^, where two different *E. faecalis* phages of the *Siphoviridae* family showed high tolerance to temperatures below 60 °C. In general, members of *Siphoviridae* family are considered to be stable at large temperature fluctuations^28^. This was the case of *E. faecalis* phage Max, as it was stable between −20 and 60 °C. For *E. faecium* phage Zip the results are similar to those observed for a *Podoviridae* phage from *Citrobacter freundii*^29^. This phage was incubated for 1 h at different temperatures showing maximum stability at 37 °C and gradually decreased the concentration as the temperature was increased up to 65 °C, being eradicated at 70 °C.

The acidity and alkalinity of the environment are also important factors influencing phage stability. Optimal pH was determined by testing the stability of phages at different pH values after 24 h of incubation at room temperature (21 °C) (Fig. 2C,D). Both phages were very stable in the pH range 5.0–11.0 and were completely inactivated at extreme pH values of 1.0, 2.0 and 13.0. For *E. faecalis* phage Max, the PFU counts decreased by 6 and 2 log CFU.mL^−1^ at pH 3.0 and 4.0, respectively (p < 0.05) (Fig. 2C). Regarding *E. faecium* phage Zip, viable phage counts were reduced in 1 log CFU.mL^−1^ at pH 4.0 and 12.0 and no phages were detected at pH 3.0 (p < 0.05) (Fig. 2D). Similarly to temperature stability results, *Siphoviridae* phages are described as the most resistant to adverse pH conditions^28^. The siphovirus phage Max showed some activity at pH 3.0, unlike the *E. faecium* podovirus Zip, inactivated at that pH value. The loss of titer at this acidic pH was already reported in the *C. freundii* phage LK1^29^.

Both phages studied in this work may be promising in combating biofilms in wounds, as they present stability in the pH range from 5–11, usually found in wounds environment. A pH value of about 4.7 is characteristic of the skin surface of adults and healthy children^30^. However, a study that analyzed 26 cases of second degree burns reported that infected wounds have an increased pH ranging from 6.5 to 9.0^31^. Moreover, concerning temperature, the skin surface temperature is about 33.2 °C^32^, but when there is an infection, the temperature in wounds can increase due to immune response, inflammatory cytokine-induced vasodilation and increased tissue metabolism^33^. Dini *et al*. analyzed the skin temperature in 18 patients affected by venous insufficiency and lower leg ulcers and described that wound injury temperature range was between 31 °C and 35 °C, and the perilesional skin temperature range was between 31 °C and 34 °C^34^.

### *Enterococcus* phage genomes are free of lysogenic genes

*Enterococcus* sp. phage genomes were sequenced through a MiSeq illumina platform and *de novo* assembled with average coverage of 324x and 230x for Max and Zip, respectively.

**Figure 3:**
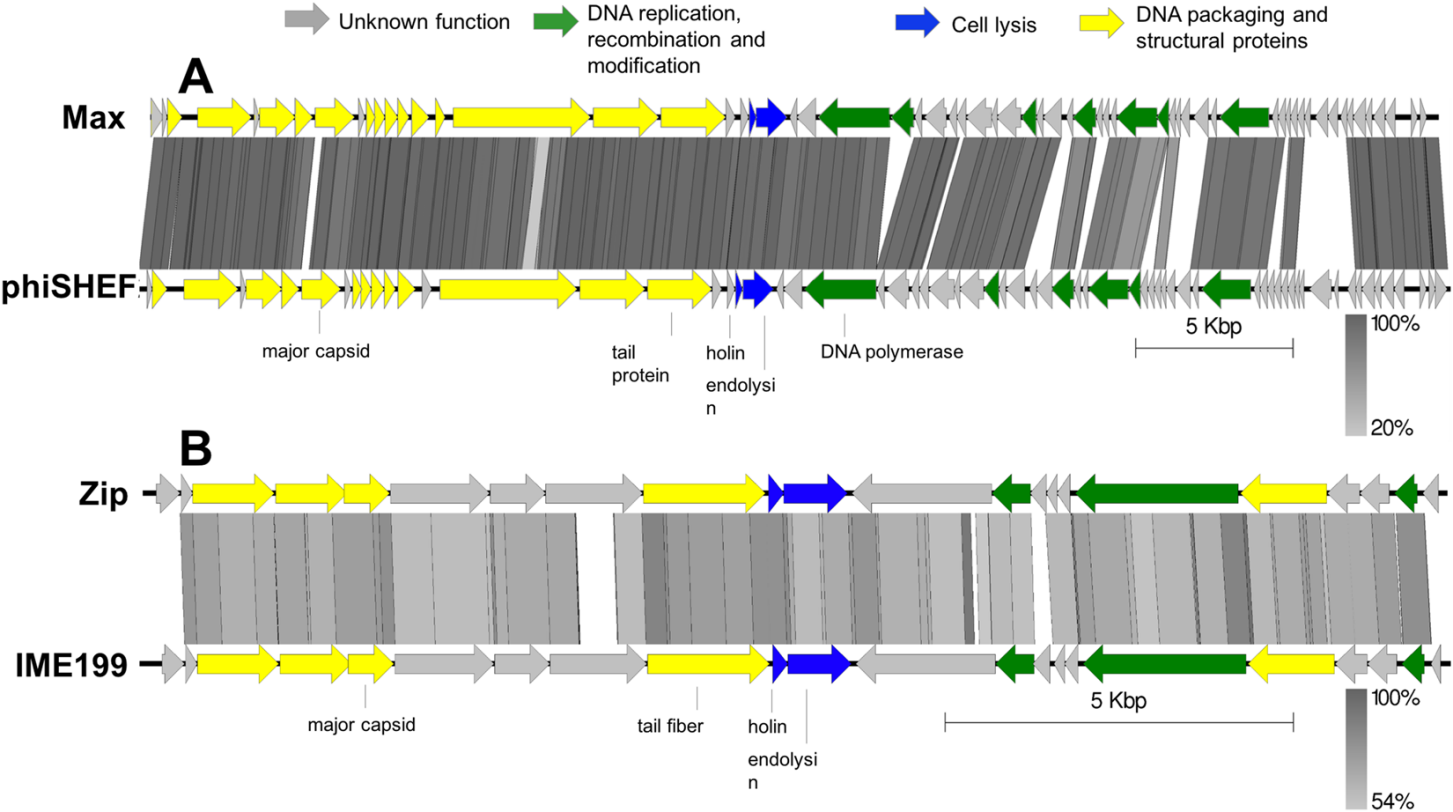
Genomic maps of enterococci phages Max and Zip. Pairwise comparisons were made to compare (**A**) Max with *Enterococcus* phage phiSHEF2 (MF678788), and (**B**) Zip with *Enterococcus* phage vB_EfaP_IME199 (KT945995), using tbBLASTX within EasyFig. Arrows indicate genes drawn to scale and coloured according to their predicted function. Gene similarity profiles between phages are indicated in grayscale (and percentage).

Max genome is a linear dsDNA molecule of 40,975 bp with a GC content of 34.7%. It encodes 65 predicted proteins in both strands being most homologs of Max found in *Enterococcus* phage genomes (Fig. 3A and Table S1). Nevertheless, only 26 proteins have assigned function to DNA replication (e.g. DNA polymerase, helicase), morphogenesis (e.g. major capsid protein, portal protein) and cell lysis (holin and endolysin). Max genome is highly syntenic with *Enterococcus* phage phiSHEF2 genome (MF678788) sharing 92% nucleotide identity and 58 of its genes. As reported for phage the similar myovirus IME-EFm1, it is expected that Max has a terminally non-redundant genome^35^. Regarding regulatory elements, we found six bacterial promoters and ten rho-independent terminators.

Zip genome has a length of 18,742 bp and GC content of 35.0%. It encodes 22 genes, of which only nine are annotated with function with relatively high identity (>84% average amino acid identity). No tRNAs or regulatory elements (promoters and terminators) were found. Annotated genes fall in three distinct functional modules: DNA packaging and structural proteins; DNA replication, recombination and modification; and cell lysis (Fig. 3B, Table S2). Comparative genomics show that Zip shares 77.4% overall nucleotide identity and all 22 genes with the *Enterococcus* phage vB_EfaP_IME199 (KT945995) (Table S2). Due to the similarities between these two genomes, it is expected that Zip has short inverted terminal repeats^22^.

Overall, the characteristics of both enterococci genomes suggest that they are safe for use in further *in vitro* studies^36^.

### Antibacterial assays reveal that Max and Zip are active against enterococci biofilms

Although the microtiter well plate model is a common method to study biofilms, mainly due to its simplicity, low price and the fact that it allows multi-parameter analysis, it has the problem of not reflecting the environmental conditions present in the wound bed^37^.

In 2010, Werthén *et al*. developed an *in vitro* model, collagen wound model (CWM), to simulate wound injury in which bacterial cells do not attach to well-defined solid surfaces, but to a collagen matrix^38^. In the referred study, it was shown that the biofilms formed in this model are structurally similar to biofilms observed in *in vivo* conditions. For this reason, CWM was already used for the study of antimicrobial activity of antibiotics and silver containing wound-dressings^39,40^.

**Figure 4:**
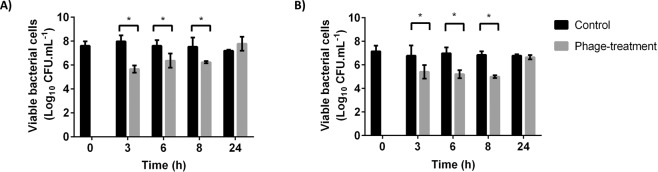
Viable bacterial cells of biofilms formed in 24-well plates using the CWM after phage infection. (**A**) Phage Max against *E. faecalis* Efa1 biofilms; (**B**) Phage Zip against *E. faecium* C410 biofilms. Error bars represent standard deviations from three independent experiments performed in duplicate. *Statistically significant (p < 0.05) conditions between control and test assays.

Herein, the efficacy of the selected phages against biofilms was tested in this model. After three hours of infection, *E. faecalis* phage Max reduced the number of viable cells in about 2 log CFU.mL^−1^ (p < 0.05) (Fig. 4A). However, at 6 h of infection, the reduction decreased to 1 log CFU.mL^−1^. Concerning *E. faecium* phage Zip, it caused a reduction of approximately 1.5 log CFU.mL^−1^ between 3 and 6 h of infection (Fig. 4B). Curiously, after 8 h the reduction increased to about 2 log CFU.mL^−1^ (p < 0.05). Although depolymerases have been described to be helpful on biofilm matrix degradation, it is currently accepted that the main function of these enzymes is the polysaccharides capsular degradation^41^. As enterococci phages Max and Zip, other phages that do not encode depolymerases have been suggested as good biofilm control agents^16,42^.

In both cases at 24 h it was observed a similar number of biofilm cells between control and phage-treated biofilms. One possible explanation for these results might be related with the emergence of phage resistance. Phage resistance was already shown to occur in other biofilm/phage interaction studies, where the proliferation of phage-resistant variants was observed for the time-points after 6 h of single-phage treatment^43^. In the same study phages revealed to be the less effective 12 h after biofilm infection, and at 48 h post-infection, no statistical differences in the number of biofilm cells between control and phage-treated biofilms, were observed. Recently, it was suggested that the combined use of phages with mechanical débridement, or other antimicrobial agents, such as antibiotics might be very helpful clinically to overcome the development of phage ressitance^15^.

Herein, both phages presented the ability to infect and kill biofilm cells in the presence of medium simulating wound injuries. However, it should be noted that although better than the microtiter plate model, CWM also possesses limitations as it is a closed system, without the presence of host immune cells and physiological factors (e.g. hypoxia, presence/absence of exudate and drainage of the wound). Nevertheless, this is a model that better mimics wounds, and our results suggest that phages Max and Zip may be promising in combating wound biofilms *in vivo*.

### The phage cocktail is active against dual-species biofilms in wound simulated conditions

The majority of the studies about chronic wounds detected more than one bacterial species on the injuries^8^. Consequently, to better simulate real biofilms, phage efficiency in mixed-*Enterococcus* strains biofilms was analyzed. The *E*. *faecalis* Efa1/*E*. *faecium* C410 consortia was studied. Competition between both species was observed as an adverse effect was detected on the *E. faecium* population. Mixed biofilms were composed by *E. faecalis* cells in about 8 log CFU.mL^−1^, a value significantly greater in 3 log CFU.mL^−1^ than the number of *E. faecium* cells. The results show that the total cells of a multi-species biofilm are not necessarily the sum of the cells of each single species. Although several researchers reported that some specific strains of enterococci can inhibit the growth of other pathogenic bacteria such as *Listeria monocytogenes*, *Clostridium tyrobutyricum* and *S. aureus*, due to the production of bacteriocins, no study has described the interactions between two *Enterococcus* specieis^44,45^. Other factors, namely competition for nutrients and the production and accumulation of toxic metabolites during biofilm formation might have contributed to these results.

**Figure 5:**
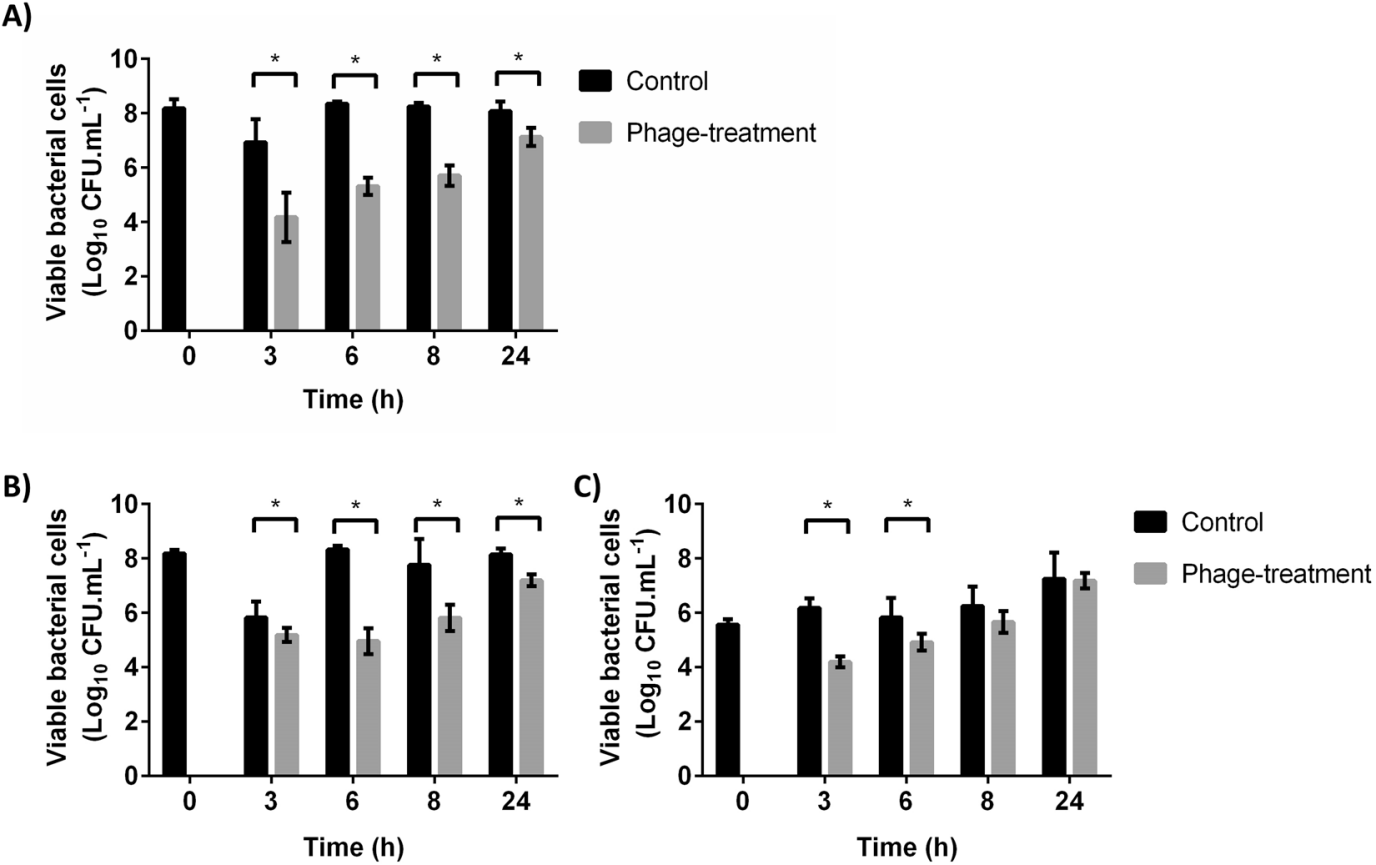
Viable bacterial cells of dual-species biofilms formed in 24-well plates using the CWM after phage cocktail infection. (**A**) Total number of viable bacterial cells; (**B**) Viable cells of *E. faecalis* Efa1; (**C**) Viable cells of *E. faecium* C410 cells. Error bars represent standard deviations from three independent experiments performed in duplicate. *Statistically significant (p < 0.05) conditions between control and test assays.

An advantage of the use of phages is that they can be mixed as cocktails to broaden collectively their antibacterial spectrum of activity^46^. In this work, a phage cocktail comprising *Enterococcus* sp phages Max and Zip was used to infect dual-species biofilms for a consortium composed by their host strains. It was decided not to apply the cocktail on single-species biofilms, due to the relevance of mixed-species biofilms. A statistically significant reduction (p < 0.05) was observed on cell concentration at 3, 6 and 8 h on treated biofilms with phage cocktail comparing to the controls (Fig. 5A). The phage cocktail was particularly efficient in reducing biofilms, where the cell concentration was reduced by approximately 2.5 log CFU.mL^−1^ after 3 h of infection (p < 0.05). The reduction remained after 6 and 8 h, although with less efficiency. As in mono-species biofilms, there is the possibility of biofilm cells might acquire resistance to phages after prolonged treatments, and consequently, after 24 h of phage infection the reduction was only of 1 log CFU.mL^−1^ (p < 0.05) (Fig. 5A). Besides the combined use of antibiotics and mechanical débridement, other possibility to avoid phage resistance relies on the use of phage cocktails comprising different phages targeting the same host, but with different receptors.

Analysis of the individual behavior of each strain shows greater reductions in the dominant strain (*E. faecalis* Efa1, Fig. 5B) than in *E. faecium* (Fig. 5C). Results suggest that the higher multiplicity of infection (MOI) applied in *E. faecium* had no positive influence on phage infection outcome. Similar observations occurred in *E. coli*, where phage JS09 was applied at different MOI and no differences were observed on phage efficiencies^47^.

The phage cocktail was more efficient in reducing biofilm cells in the CWM than any single phage administration in mono-species biofilms. Similar results were observed in an *in vivo* wound model where the therapeutic efficacy of *Klebsiella pneumoniae* phages was evaluated in comparison to the phage cocktail in the treatment of wound infection. Mice receiving phage cocktail treatment showed a decrease of 6 log CFU.mL^−1^ in bacterial load as compared to the control, while for single phage treatment a decrease of about 4.5 log CFU.mL^−1^ was observed^48^.

### Max and Zip are not cytotoxic to epithelial cells

**Figure 6:**
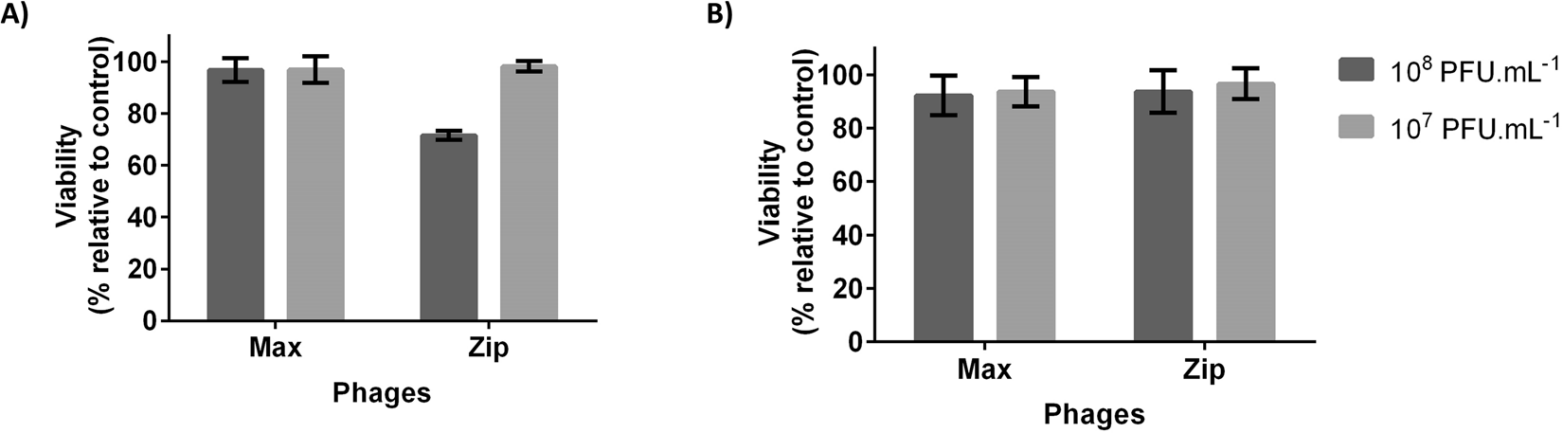
Viability values (%) of 3T3 cells exposed to different concentrations of *E. faecalis* phage Max and *E. faecium* phage Zip for 24 h (**A**) Phage lysate; (**B**) Phages purified by PEG/NaCl precipitation. Viability was calculated as percentage of negative control (3T3 cells without phages). Error bars represent standard deviations from three independent experiments performed in duplicate.

Cytotoxicity assays were performed using *E. faecalis* phage Max and *E. faecium* phage Zip. These phages were tested in two different concentrations (10^8^ and 10^7^ PFU.mL^−1^) and in two different conditions - lysate and after PEG purification. Several assays are available to measure cell cytotoxicity, but no standard assay is described for determining the cytotoxic effect of phages^49^. In general, both phages were nontoxic to 3T3 cells in all conditions tested (viability of cells was about 100%) (Fig. 6A,B). Nevertheless, the lysate of phage Zip at 10^8^ PFU.mL^−1^ showed some toxicity, causing a loss of about 30% of cells (Fig. 6A). It is possible that some components of the lysate, originating from the bacteria, have some cytotoxic effect on the cells, which disappears after PEG purification, demonstrating the phage itself is not toxic.

Other studies on phage cytotoxicity in mammalian cells have shown that phages have no effect on cell viability. For example, Merabishvili *et al*. concluded that a phage cocktail active against *P. aeruginosa* and *S. aureus* strains was not cytotoxic against human neonatal foreskin keratinocytes^50^. Although several *in vivo* studies reported the safety of phages, similar studies should be performed for every new isolated phage that could be used for therapeutic purposes.

### Max and Zip are active against bacteria colonizing 3T3 cells

We also studied the behavior of phages against bacterial infections in animal cells by investigating the mechanisms by which bacteria and phages interact with these cells.

**Figure 7:**
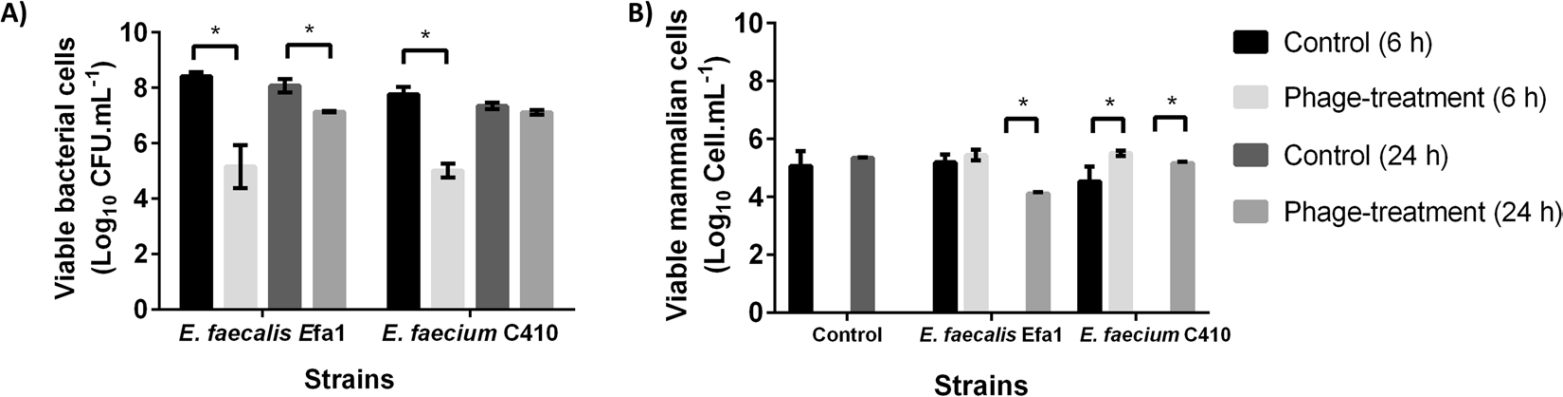
Efficacy of phages against bacteria colonizing 3T3 cells. (**A**) Concentration of viable bacterial cells; and (**B**) number of 3T3 cells in control (without treatment) and after 6 and 24 h of phage treatment. Error bars represent standard deviations from three independent experiments performed in duplicate. *Statistically significant (p < 0.05) conditions between control and test assays.

3T3 cells were infected with 10^8^ CFU.mL^−1^ of each bacterium (*E. faecalis* Efa1 and *E. faecium* C410) and treated at 2 h post-infection with 10^7^ PFU.mL^−1^ of the respective phage. The concentration of viable bacterial cells and the number of 3T3 cells were quantified at 6 and 24 h post-treatment (Fig. 7). Concerning the reduction of infection, both tested phages caused a decrease in viable bacterial cells (p < 0.05) after 6 h of treatment (Fig. 7A). The reduction was of about 3 log CFU.mL^−1^, which was higher than the reduction previously obtained on biofilms. In 3T3 cells, 6 h after the application of *E. faecium* phage Zip, it was possible to observe a greater concentration (1 log CFU.mL^−1^, p < 0.05) of viable 3T3 cells than in the untreated control cells (Fig. 7B). In the other cases at 6 h of phage treatment, the concentration of 3T3 cells was about 5.5 log CFU.mL^−1^, very similar between treated and untreated cells. Twenty-four hours after phage treatment, it was possible to observe that only phage-treated epithelial cells were still viable (Fig. 7B). Curiously, at this time point, phage efficacy in the reduction of bacterial cells was very reduced (between 0.2 and 1 log CFU.mL^−1^, Fig. 7A), which demonstrates the long-lasting beneficial effect of phage treatment on mammalian cell viability. A possible explanation for mammalian cell survival might be related with the reduced virulence of phage resistant phenotypes. It has been suggested that the selective pressure exerted by phages can lead to the proliferation of less virulent strains^51^. Moreover, in real infective conditions, the immune system can help phages to eliminate the surviving bacterial cells^52^.

The efficacy of phages against bacteria adhered to mammalian cells was observed by Mirzaei *et al*., when they studied the interaction between immortalized cell lines (HT-29 and Caco-2 intestinal epithelial cells) and four *E. coli* phages. In that study, phages showed a significant reduction between 1.5 and 4.5 log CFU.mL^−1^ of the bacterial content over a period of 8 h^53^. Although the data about the effects of direct addition of phages against bacteria in immortalized cell lines is scarce, phages efficacy on *in vivo* models suggests that this approach might be a valuable therapy.

This work showed that the two phages can infect bacteria that colonized epithelial cells, suggesting its therapeutic interest.

## Conclusions

The properties demonstrated by the novel isolated phages, namely broad lytic spectra, stability at different temperatures and pH ranges, lack of lysogenic content on their genomes, efficacy against biofilms in simulated wound conditions and against colonized epithelial cells suggest their therapeutic use. Further studies should be performed to confirm the *in vivo* efficacy of single or combined application of the phages.

## Methods

### Bacterial strains and culture conditions

A total of 16 *E. faecalis* and 13 *E. faecium* clinical isolates from Hospital de Braga and collection strains were used for phages isolation (Table 1). To complete the analysis of the phages lytic spectra, one *E. gallinarum* strain and additional Gram-positive species from our collection were used, including three staphylococci strains and one representative of *Listeria monocytogenes* (Table 1). All strains were grown in Tryptic Soy Broth (TSB), Tryptic Soy Agar (TSA) or in TSA soft overlays (TSB with 0.6% agar) at 37 °C.

### Phage isolation and production

Phage isolation was performed as previously described^54^. Wastewaters from ETAR Frossos, Braga, were centrifuged (8 500 × g, 4 °C, and 10 min) and the supernatant collected. Then, 50 mL of sewage supernatant were mixed with the same volume of double-strength TSB and 100 μL of each bacterial suspension grown overnight. The mixtures were further incubated for 24 h, 120 rpm, at 37 °C. The culture was centrifuged (10 000 × g, 4 °C, 10 min) and the supernatant filtered (0.22 μm). Spot assays were performed on bacterial lawns to check the presence of phages. Inhibition zones (clear or turbid), indicative of the presence of phages were further purified with toothpicks and paper to isolate all different phages. Plaque picking was repeated until single-plaque morphology was observed.

Each isolated phage was produced as previously described^55^. Briefly, a phage plaque of each phage was picked with a toothpick and stung in agar plates containing a bacterial lawn. A strip of paper was passed through the entire agar plates, and the plates were incubated overnight at 37 °C.

After full lysis, 3 mL of SM buffer (5.8 g.L^−1^ NaCl, 2 g.L^−1^ MgSO4.7H2O, 50 mL.L^−1^ 1 M Tris pH 7.5, 0.002% (w/v) gelatin) was added to each plate. Plates were further incubated at 4 °C, 50–90 rpm, for 7 h. The liquid and top-agar with the eluted phages were collected and the solution was centrifuged (5 000 × g, 4 °C, 5 min) to remove all remaining bacteria. The supernatant was recovered and filtered (0.22 μm).

Phage particles were further purified as previously described by Sambrook *et al*.^56^. Sodium chloride (0.584 g/10 mL) was added to the phage lysate and the solution was incubated at 4 °C, 50–90 rpm, for 1 h. The solution was centrifuged (8 500 × g, 4 °C, and 10 min) and the supernatant was recovered. Then PEG 8000 (1 g/10 mL) was added and gently mixed until dissolved, and further incubated overnight at 4 °C, 50–90 rpm. The solution was then centrifuged (8 500 × g, 4 °C, and 10 min), and the pellet was recovered and eluted in SM buffer at 4 °C, 50–90 rpm, for 1 h. 25% (v/v) chloroform was added and mixed, and the solution centrifuged (3 500 × g, 4 °C, and 15 min). The top aqueous layer containing the purified phages was recovered, filtered (0.22 μm), and stored at 4 °C for later use.

Phage titration was performed according to the double agar overlay technique^57^. Briefly, 100 μL of serially diluted phage solution, 100 μL of host bacteria culture and 3 mL of soft agar were mixed and poured onto a TSA plate. After overnight incubation at 37 °C, the plaque forming units (PFUs) were determined.

### Electron microscopy

The morphology of phage particles was observed by TEM, as previously described^58^. Briefly, a solution of phage lysate was centrifuged (1 h, 25 000 × *g*, 4 °C) and phage particles were collected. The pellet was washed twice in tap water using the same centrifugation conditions. Phages were further deposited on copper grids with carbon-coated Formvar films and stained with 2% uranyl acetate (pH 4.0). Phages were examined using a Jeol JEM 1400 transmission electron microscope.

### Determination of the host range

The host range of phages Max and Zip was determined as previously described with some modifications^59^. A total of 33 strains were selected for determination of host range (Table 1). Bacterial lawns were made on TSA plates by adding 100 μL of exponential-phase cell cultures of each strain. Then, 10 μL of each phage solution at approximately 10^7^ PFU.mL^−1^ were applied to the plate and incubated at 37 °C for 16–18 h to observe whether there were transparent regions on the plate.

### Thermal and pH stability

Thermal stability tests were carried essentially as previously described^60^. A phage titer of 10^8^ PFU.mL^−1^ of each tested phage was incubated at −20 °C, 4 °C (as control), 21 °C (room temperature), 37 °C, 50 °C and 60 °C for 24 h. Similarly, the effect of pH was also evaluated using a universal pH buffer (150 mM potassium chloride, 10 mM potassium dihydrogen phosphate, 10 mM sodium citrate, 10 mM boric acid with pH adjusted to 1, 2, 3, 4, 5, 6, 7 (as control), 8, 9, 10, 11, 12 and 13, at room temperature (21 °C) for 24 h. In both experiments, concentration of phage in PFU.mL^−1^ was determined by equation of phage enumeration. Three experiments were performed in duplicate.

### DNA isolation, genome sequencing and *in silico* analysis

Phage genomic DNA was isolated using the phenol-chloroform-isoamyl alcohol method as described elsewhere^58^. The DNA samples were used for library construction using the Illumina Nextera XT library preparation kit. The generated DNA fragments (DNA libraries) were sequenced in the lllumina MiSeq platform, using 250 bp paired-end sequencing reads. The raw sequence data underwent automatic initial treatment, namely adapters and low quality bases trimming. Demultiplexed reads were *de novo* assembled into a single contig using Geneious R9. The assembled genomes were scanned through MyRAST^61^, tRNAscan-SE^62^ and BLASTP^63^ to search for coding regions, tRNAs and find protein homologs, respectively. Proteins sequenced were analyzed with TMHMM^64^ and SignalP^65^ to predict transmembrane domains and signal peptide cleavage sites. Comparative genomics was performed with BLASTN or OrthoVenn^66^. Genomes were visualized with Easyfig.^67^. The complete genome sequence of phages Max and Zip have been deposited in the NCBI database under accession no. MK360024 and MK360025, respectively.

### Biofilm formation using the collagen wound model

The CWM for biofilm formation was used as described by Werthén *et al*.^38^. Two mL of collagen from bovine Achilles tendon (50 μg.mL^−1^ suspended in 0.9% NaCl), were added to each well of 24-well plates (Orange Scientific) and the plates were incubated overnight at 4 °C. The coating solution was then gently removed and the wells were washed twice with 0.9% NaCl prior to biofilm formation.

Single-species biofilms were formed by mixing of bacterial suspensions (grown overnight at 37 °C and 120 rpm) and a mixture of TSB/SWF (1:1) culture medium (SWF: 50% Fetal Bovine Serum (FBS) and 50% physiological NaCl in 0.1% Pepton) at a approximate concentration of 2 × 10^7^ CFU.mL^−1^. For dual-species biofilms, 5 μL of each bacterial culture were added to each well. Plates were further incubated at 37 °C and 120 rpm for 48 h, with complete culture media renewal after 24 h. In negative control wells, only TSB/SWF (1:1) medium was added. After biofilm formation, wells were washed twice with 0.9% NaCl, to remove planktonic cells. Three experiments were performed in duplicate.

### Biofilm infection

After single-species biofilm formation, a phage solution with 2 × 10^8^ PFU.mL^−1^ was added to each well. On dual-species biofilms a cocktail containing 1 × 10^8^ PFU.mL^−1^ of each phage was added to each well. Then, the plates were incubated at 37 °C and samples were taken at 3, 6, 8 and 24 h to determine the number of cultivable bacteria. At each time point, biofilms formed in each well, were disrupted using an ultrasonic bath operating at 50 kHz for 30 min, and then the suspensions were collected and viable cells were determined by CFU counting. For counting of specific strains, CFUs were determined under selective conditions, by diluting the bacteria in the presence of the phage active against the opponent species. Three experiments were performed in duplicate.

### Cytotoxicity assays

Mouse embryonic fibroblast 3T3 cell line was obtained from the American Type Culture Collection (ATCC). Cells were grown in Dulbecco’s Modified Eagle’s Medium (DMEM), supplemented with 10% (v/v) FBS, 1% (v/v) of penicillin-streptomycin (ThermoFisher) (complete culture medium), on tissue culture-treated flasks, at 37 °C, 5% CO_2_ and >90% humidity. Sub-culturing was performed when cell confluence reached approximately 80%, at a 1:2 or 1:3 flask ratio. For this, cells were washed with Phosphate Buffered Saline (PBS: 137 mM NaCl, 10 mM Sodium Phospate Dibasic, 2.7 mM Potassium Chloride and Potassium Phosphate Monobasic, pH = 7.4) and detached using trypsin-EDTA.

The cytotoxicity of the two phages was determined according to the ISO 10993-5:2009, Annex A - Neutral Red Uptake (NRU) cytotoxicity test. Neutral red (NR) is a weak cationic dye used to identify viable cells in culture. This assay consists on the uptake of NR into lysosomes and its subsequent accumulation on living cells, indicating quantitative viability. If cells are damaged or dead, NR is no longer retained within the vacuole of cells. Thus, the NR viability assay quantifies the number of viable, uninjured cells after their exposure with test agents, through reduction in the absorbance^68^. Briefly, 3T3 cells were seeded into 96-well plates, at 1 × 10^5^ cells.mL^−1^ in complete culture medium. Plates were maintained in the aforementioned culture conditions.

After 24 h incubation, the culture medium was removed and 100 μL of treatment medium, composed of complete culture medium containing the appropriate concentrations of phages (10^8^ PFU.mL^−1^ and 10^7^ PFU.mL^−1^) was added. A positive control consisting of complete culture medium containing different concentrations (0.20, 0.15, 0.10 and 0.05 mg.mL^−1^) of sodium lauryl sulfate (SLS, ThermoFisher) was also added to the cells. A negative control was prepared with complete culture medium, and a blank with culture medium without 3T3 cells. The 96-well plates containing these preparations were incubated for 24 h in the conditions described previously.

In the third day, the culture medium was removed and the cells were washed with pre-warmed PBS. The stock solution of NR (3-amino-m-dimethylamino-2-methlphenazine hydrochloride) was prepared using 0.4% of NR stock in 100 mL of sterile water, which was then mixed with DMEM (to a final concentration of 1.25% (v/v)). Then 100 μL of this solution were added to each well and incubated at 37 °C and 5% of CO_2_, in a humidified atmosphere, for 3 h. The NR solution was removed and the cells were washed with pre-warmed PBS. Subsequently, 150 μL of NR desorb solution (1% acetic acid, 50% ethanol and 49% sterile water) were added to all wells and the plates were shaken for 10 min, to extract the NR from the cells and form a homogeneous solution. NR absorption was measured at an optical density of 540 nm (OD_540_) in a spectrophotometer. To analyze cell viability, the NR absorbance of the sample was divided by the NR absorbance of the negative control. The assays were performed in triplicate, and considered valid only when the IC50 of SDS was within the confidence interval of 0.070–0.116 mg.mL^−1^ (95% confidence) and blank OD_540_ was equal or greater than 0.3. Three experiments were performed in duplicate.

### Efficacy of phages against bacteria colonizing 3T3 cells

Two culture media were used in this assay: Culture medium 1, DMEM supplemented with 10% (v/v) FBS and 1% (v/v) penicillin-streptomycin; and culture medium 2, DMEM supplemented with 10% (v/v) FBS. 3T3 cells were seeded into 96-well plates at 5 × 10^5^ cells.mL^−1^ on culture medium 1, and plates were maintained at growing conditions for 24 h.

Bacterial suspensions grown overnight in TSB medium were centrifuged (11 000 × g, 1 min), washed twice with PBS, and the OD_600_ nm adjusted to 0.08 with PBS. Bacterial cells were further centrifuged and re-suspended in culture medium 2. Then, 100 μL of the bacterial suspensions at a concentration of approximately 1 × 10^8^ CFU.mL^−1^ were added to the 3T3 cells in the 96-well plates, previously washed with PBS. Negative control was prepared with culture medium 2. The 96-well plates containing these preparations were incubated for 2 h.

Phage treatment was performed on cells colonized by bacteria after 2 h. Briefly, 100 μL of phage solutions at 1 × 10^7^ PFU.mL^−1^ were added to the cells, and 100 μL of culture medium 2 were added to negative and non-phage treated cells. The 96-well plates containing these preparations were incubated for 6 h or 24 h at growing conditions.

Afterwards, the culture medium was removed and cells were washed twice with PBS. Then, 40 μL of pre- warmed trypsin were added to all wells, and the plates were incubated for 15 min at growing conditions. To stall trypsin activity, 60 μL of culture medium 2 were added to each well. The CFU method was used for the determination of viable bacterial cell concentration. Mammalian cell death associated with bacterial adhesion was quantified with a Neubauer chamber and an inverted microscope. Three experiments were performed in duplicate.

### Statistical analysis

Statistical analysis was carried out by two-way repeated-measures analysis of variance (ANOVA) with Bonferroni post hoc tests. Differences between samples were considered statistically different for p-values lower than 0.05.

## Supplementary information

Table S1; Table S2

## Data Availability

All data generated or analyzed during this study are included in this published article and its Supplementary Information files.

## References

[1] Clark RA, Ghosh K, Tonnesen MG (2007). Tissue engineering for cutaneous wounds. The Journal of investigative dermatology.

[2] Frykberg RG, Banks J (2015). Challenges in the Treatment of Chronic. Wounds. Advances in wound care.

[3] Jarbrink K (2017). The humanistic and economic burden of chronic wounds: a protocol for a systematic review. Systematic reviews.

[4] Sen CK (2009). Human skin wounds: a major and snowballing threat to public health and the economy. Wound repair and regeneration: official publication of the Wound Healing Society [and] the European Tissue Repair Society.

[5] Parsek MR, Singh PK (2003). Bacterial biofilms: an emerging link to disease pathogenesis. Annual review of microbiology.

[6] Attinger C, Wolcott R (2012). Clinically Addressing Biofilm in Chronic Wounds. Advances in wound care.

[7] Wolcott RD, Rhoads DD, Dowd SE (2008). Biofilms and chronic wound inflammation. Journal of wound care.

[8] Gjodsbol K (2006). Multiple bacterial species reside in chronic wounds: a longitudinal study. International wound journal.

[9] Tzaneva V, Mladenova I, Todorova G, Petkov D (2016). Antibiotic treatment and resistance in chronic wounds of vascular origin. Clujul medical.

[10] James GA (2008). Biofilms in chronic wounds. Wound repair and regeneration: official publication of the Wound Healing Society [and] the European Tissue Repair Society.

[11] Bowler PG, Duerden BI, Armstrong DG (2001). Wound microbiology and associated approaches to wound management. Clinical microbiology reviews.

[12] Cataldo MC (2011). Colonization of pressure ulcers by multidrug-resistant microorganisms in patients receiving home care. Scandinavian journal of infectious diseases.

[13] Arias CA, Murray BE (2012). The rise of the *Enterococcus*: beyond vancomycin resistance. Nature reviews. Microbiology.

[14] Organization, W. W. H. WHO publishes list of bacteria for which new antibiotics are urgently needed. (2017).

[15] Pires DP, Melo L, Vilas Boas D, Sillankorva S, Azeredo J (2017). Phage therapy as an alternative or complementary strategy to prevent and control biofilm-related infections. Current opinion in microbiology.

[16] Khalifa L (2015). Targeting *Enterococcus faecalis* biofilms with phage therapy. Applied and environmental microbiology.

[17] Khalifa L (2018). Defeating Antibiotic- and Phage-Resistant *Enterococcus faecalis* Using a Phage Cocktail *in Vitro* and in a Clot Model. Frontiers in microbiology.

[18] Tinoco JM (2016). Effect of a genetically engineered bacteriophage on *Enterococcus faecalis* biofilms. Archives of oral biology.

[19] Chibani-Chennoufi S, Bruttin A, Dillmann ML, Brussow H (2004). Phage-host interaction: an ecological perspective. Journal of bacteriology.

[20] Ackermann HW (1998). Tailed bacteriophages: the order caudovirales. Advances in virus research.

[21] Zhang W (2013). Characterization of *Enterococcus faecalis* phage IME-EF1 and its endolysin. PloS one.

[22] Xing S (2017). Complete genome sequence of a novel, virulent *Ahjdlikevirus* bacteriophage that infects *Enterococcus faecium*. Archives of virology.

[23] Casey Eoghan, van Sinderen Douwe, Mahony Jennifer (2018). In Vitro Characteristics of Phages to Guide ‘Real Life’ Phage Therapy Suitability. Viruses.

[24] Gong P (2016). Characterization of *Enterococcus faecium* bacteriophage IME-EFm5 and its endolysin LysEFm5. Virology.

[25] Jonczyk E, Klak M, Miedzybrodzki R, Gorski A (2011). The influence of external factors on bacteriophages–review. Folia microbiologica.

[26] Olson MR, Axler RP, Hicks RE (2004). Effects of freezing and storage temperature on MS2 viability. Journal of virological methods.

[27] Lee Y-D, Chun H, Park J-H (2014). Characteristics and growth inhibition of isolated bacteriophages for *Enterococcus faecalis*. Food Science and Biotechnology.

[28] Lasobras J, Muniesa M, Frías J, Lucena F, Jofre J (1997). Relationship between the morphology of bacteriophages and their persistence in the environment. Water Science and Technology.

[29] Chaudhry WN, Haq IU, Andleeb S, Qadri I (2014). Characterization of a virulent bacteriophage LK1 specific for *Citrobacter freundii* isolated from sewage water. Journal of basic microbiology.

[30] Lambers H, Piessens S, Bloem A, Pronk H, Finkel P (2006). Natural skin surface pH is on average below 5, which is beneficial for its resident flora. International journal of cosmetic science.

[31] Ono S (2015). Increased wound pH as an indicator of local wound infection in second degree burns. Burns: journal of the International Society for Burn Injuries.

[32] Karlsbad A, Kopp S (1991). Intramuscular and skin surface temperatures of the resting human superficial masseter muscle. Acta odontologica Scandinavica.

[33] Power G, Moore Z, O’Connor T (2017). Measurement of pH, exudate composition and temperature in wound healing: a systematic review. Journal of wound care.

[34] Dini V (2015). Correlation Between Wound Temperature Obtained With an Infrared Camera and Clinical Wound Bed Score in Venous Leg Ulcers. Wounds: a compendium of clinical research and practice.

[35] Wang Y (2014). Characterization and complete genome sequence analysis of novel bacteriophage IME-EFm1 infecting *Enterococcus faecium*. The Journal of general virology.

[36] Philipson Casandra, Voegtly Logan, Lueder Matthew, Long Kyle, Rice Gregory, Frey Kenneth, Biswas Biswajit, Cer Regina, Hamilton Theron, Bishop-Lilly Kimberly (2018). Characterizing Phage Genomes for Therapeutic Applications. Viruses.

[37] Azeredo J (2017). Critical review on biofilm methods. Critical reviews in microbiology.

[38] Werthen M (2010). An *in vitro* model of bacterial infections in wounds and other soft tissues. APMIS: acta pathologica, microbiologica, et immunologica Scandinavica.

[39] Brackman G, Cos P, Maes L, Nelis HJ, Coenye T (2011). Quorum sensing inhibitors increase the susceptibility of bacterial biofilms to antibiotics *in vitro* and *in vivo*. Antimicrobial agents and chemotherapy.

[40] Hakonen B, Lonnberg LK, Larko E, Blom K (2014). A Novel Qualitative and Quantitative Biofilm Assay Based on 3D Soft Tissue. International journal of biomaterials.

[41] Pires DP, Oliveira H, Melo LD, Sillankorva S, Azeredo J (2016). Bacteriophage-encoded depolymerases: their diversity and biotechnological applications. Applied microbiology and biotechnology.

[42] Vandersteegen K (2013). Romulus and Remus, two phage isolates representing a distinct clade within the *Twortlikevirus* genus, display suitable properties for phage therapy applications. Journal of virology.

[43] Pires D, Sillankorva S, Faustino A, Azeredo J (2011). Use of newly isolated phages for control of *Pseudomonas aeruginosa* PAO1 and ATCC 10145 biofilms. Research in microbiology.

[44] Belgacem ZB (2010). Antimicrobial activity, safety aspects, and some technological properties of bacteriocinogenic *Enterococcus faecium* from artisanal Tunisian fermented meat. Food Control.

[45] da Silva Fernandes M, Kabuki DY, Kuaye AY (2015). Behavior of *Listeria monocytogenes* in a multi-species biofilm with *Enterococcus faecalis* and *Enterococcus faecium* and control through sanitation procedures. International journal of food microbiology.

[46] Chan BK, Abedon ST, Loc-Carrillo C (2013). Phage cocktails and the future of phage therapy. Future microbiology.

[47] Zhou Y, Bao H, Zhang H, Wang R (2015). Isolation and Characterization of Lytic Phage vB_EcoM_JS09 against Clinically Isolated Antibiotic-Resistant Avian Pathogenic *Escherichia coli* and Enterotoxigenic *Escherichia coli*. Intervirology.

[48] Chadha P, Katare OP, Chhibber S (2016). *In vivo* efficacy of single phage versus phage cocktail in resolving burn wound infection in BALB/c mice. Microbial pathogenesis.

[49] Henein AE, Hanlon GW, Cooper CJ, Denyer SP, Maillard JY (2016). A Partially Purified *Acinetobacter baumannii* Phage Preparation Exhibits no Cytotoxicity in 3T3 Mouse Fibroblast. Cells. Frontiers in microbiology.

[50] Merabishvili M (2009). Quality-controlled small-scale production of a well-defined bacteriophage cocktail for use in human clinical trials. PloS one.

[51] Leon M, Bastias R (2015). Virulence reduction in bacteriophage resistant bacteria. Frontiers in microbiology.

[52] Roach DR (2017). Synergy between the Host Immune System and Bacteriophage Is Essential for Successful Phage Therapy against an Acute Respiratory Pathogen. Cell host & microbe.

[53] Khan Mirzaei M (2016). Morphologically Distinct *Escherichia coli* Bacteriophages Differ in Their Efficacy and Ability to Stimulate Cytokine Release. In Vitro. Frontiers in microbiology.

[54] Melo LD (2014). Characterization of *Staphylococcus epidermidis* phage vB_SepS_SEP9 - a unique member of the *Siphoviridae* family. Research in microbiology.

[55] Melo LD (2016). Development of a Phage Cocktail to Control *Proteus mirabilis* Catheter-associated Urinary Tract Infections. Frontiers in microbiology.

[56] Sambrook, J. & Russell, D. W. *Molecular Cloning: A Laboratory Manual, 3rd ed*. (Cold Spring Harbor Laboratory Press, 2001).

[57] Kropinski AM, Mazzocco A, Waddell TE, Lingohr E, Johnson RP (2009). Enumeration of bacteriophages by double agar overlay plaque assay. Methods in molecular biology.

[58] Melo LD (2014). Isolation and characterization of a new *Staphylococcus epidermidis* broad-spectrum bacteriophage. The Journal of general virology.

[59] Danis-Wlodarczyk K (2015). Characterization of the Newly Isolated Lytic Bacteriophages KTN6 and KT28 and Their Efficacy against *Pseudomonas aeruginosa* Biofilm. PloS one.

[60] Oliveira H (2016). Characterization and genome sequencing of a *Citrobacter freundii* phage CfP1 harboring a lysin active against multidrug-resistant isolates. Applied microbiology and biotechnology.

[61] Aziz RK (2008). The RAST Server: rapid annotations using subsystems technology. BMC genomics.

[62] Schattner P, Brooks AN, Lowe TM (2005). The tRNAscan-SE, snoscan and snoGPS web servers for the detection of tRNAs and snoRNAs. Nucleic acids research.

[63] Altschul SF, Gish W, Miller W, Myers EW, Lipman DJ (1990). Basic local alignment search tool. Journal of molecular biology.

[64] Krogh A, Larsson B, von Heijne G, Sonnhammer EL (2001). Predicting transmembrane protein topology with a hidden Markov model: application to complete genomes. Journal of molecular biology.

[65] Petersen TN, Brunak S, von Heijne G, Nielsen H (2011). SignalP 4.0: discriminating signal peptides from transmembrane regions. Nature methods.

[66] Wang Y, Coleman-Derr D, Chen G, Gu YQ (2015). OrthoVenn: a web server for genome wide comparison and annotation of orthologous clusters across multiple species. Nucleic acids research.

[67] Sullivan MJ, Petty NK, Beatson SA (2011). Easyfig: a genome comparison visualizer. Bioinformatics.

[68] Todaro GJ, Green H (1963). Quantitative studies of the growth of mouse embryo cells in culture and their development into established lines. The Journal of cell biology.

