# Author Correction: Efficacy and safety assessment of two enterococci phages in an in vitro biofilm wound model

**DOI:** 10.1038/s41598-021-90464-4

**Published:** 2021-05-20

**Authors:** Luís D. R. Melo, R. Ferreira, Ana R. Costa, H. Oliveira, J. Azeredo

**Affiliations:** CEB - Centre of Biological Engineering, University of Minho, 4710-057 Braga, Portugal

Correction to: *Scitific Reports* 10.1038/s41598-019-43115-8, published online 30 April 2019

The original version of this Article contained an error in the Acknowledgements section.

“This study was supported by the Portuguese Foundation for Science and Technology (FCT) under the scope of the strategic funding of UID/BIO/04469/2013 unit, COMPETE 2020 (POCI-01-0145-FEDER-006684) and the Project PTDC/BBB-BSS/6471/2014 (POCI-01-0145-FEDER-016678). This work was also supported by BioTecNorte operation (NORTE-01-0145-FEDER-000004) funded by the European Regional Development Fund under the scope of Norte2020 - Programa Operacional Regional do Norte. A.R.C. and H.O. acknowledge FCT for grant SFRH/BPD/94648/2013 and SFRH/BPD/111653/2015, respectively. The funders had no role in study design, data collection and analysis, decision to publish, or preparation of the manuscript.”

now reads:

“This study was supported by the Portuguese Foundation for Science and Technology (FCT) under the scope of the strategic funding of UID/BIO/04469/2013 unit, COMPETE 2020 (POCI-01-0145-FEDER-006684) and the Project PTDC/BBB-BSS/6471/2014 (POCI-01-0145-FEDER-016643). This work was also supported by BioTecNorte operation (NORTE-01-0145-FEDER-000004) funded by the European Regional Development Fund under the scope of Norte2020 - Programa Operacional Regional do Norte. A.R.C. and H.O. acknowledge FCT for grant SFRH/BPD/94648/2013 and SFRH/BPD/111653/2015, respectively. The funders had no role in study design, data collection and analysis, decision to publish, or preparation of the manuscript.”

The original Article has been corrected.

