# Supplementary material for: Efficacy and safety assessment of two enterococci phages in an in vitro biofilm wound model
Source: Sci Rep. 2019 Apr 30;9:6643. doi: 10.1038/s41598-019-43115-8 (PMC6491613; doi:10.1038/s41598-019-43115-8)
Supplement: Supplementary file 1 — Table S1; Table S2 [file 41598_2019_43115_MOESM1_ESM.pdf]

Efficacy and safety assessment of two enterococci phages in an *in vitro* biofilm wound model

Luís D. R. Melo, R. Ferreira, Ana R. Costa, H. Oliveira, J. Azeredo*

CEB – Centre of Biological Engineering, University of Minho, 4710-057, Braga, Portugal.

* Corresponding author:

Joana Azeredo,

Centre of Biological Engineering

Campus de Gualtar, 4710-057 Braga, Portugal

Telephone: (+351) 253 604 424; e-mail: jazeredo@deb.uminho.pt

Table S1. Genomic features of the *Enterococcus faecalis* phage Max.

			**Predicted protein**						
**ORF (+/-)**	**Start (bp)**	**Stop (bp)**	**Size (aa)**	**MW (kDa)**	**pI**	**Best species hit**	**Putative function**	**E-value (% identify)**	**Signals/Motifs**
1/+	18	416	132	15.15	10.26	*Enterococcus* phage EfaCPT1	hypothetical protein	7,00E-85 (94%)	-
2/+	376	528	50	5.96	10.40	*Enterococcus* phage EFAP-1	hypothetical protein	7,00E-29 (100%)	-
3/+	543	1,016	157	19.11	4.92	*Enterococcus* phage EFAP-1	terminase small subunit	3,00E-112 (100%)	-
4/+	1,501	3,225	574	65.80	5.12	*Enterococcus* phage IME-EF4	terminase large subunit	0 (99%)	-
5/+	3,295	3,459	54	5.79	3.98	*Enterococcus* phage IME_EF3	hypothetical protein	1,00E-27 (100%)	2 TMD
6/+	3,464	4,615	383	42.94	4.75	*Enterococcus* phage EfaCPT1	portal protein	0 (99%)	1 TMD
7/+	4,602	5,165	187	21.13	4.99	*Enterococcus* phage vB_EfaS_IME196	head maturation protease	1,00E-131 (99%)	-
8/+	5,236	6,489	417	46.91	4.43	*Enterococcus* phage EfaCPT1	major capsid protein	0,00E+00 (98%)	-
9/+	6,615	6,815	66	6.98	6.74	*Enterococcus* phage EFRM31	hypothetical protein	1,00E-32 (86%)	-
10/+	6,859	7,155	98	11.23	4.64	*Enterococcus* phage EFRM31	head-tail joining protein	3,00E-64 (99%)	-
11/+	7,127	7,462	111	13.39	8.20	*Enterococcus* phage AUEF3	head-tail joining family protein	7,00E-76 (98%)	-
12/+	7,459	7,866	135	15.37	9.64	*Enterococcus* phage EFRM31	minor capsid protein	1,00E-94 (99%)	-
13/+	7,863	8,228	121	14.20	4.41	*Enterococcus* phage EFRM31	head-tail joining protein	4,00E-85 (100%)	-
14/+	8,304	8,870	188	19.76	4.28	*Enterococcus* phage EfaCPT1	major tail tube protein	4,00E-129 (99%)	-
15/+	9,065	9,376	103	11.64	4.21	*Enterococcus* phage EfaCPT1	tail tape measure chaperone protein	1,00E-67 (100%)	-
16/+	9,633	14,003	1456	156.46	10.26	*Enterococcus* phage EfaCPT1	tail tape measure protein	0 (98%)	-
17/+	14,086	16,167	693	79.66	4.88	*Enterococcus* phage AUEF3	tail protein	0 (96%)	-
18/+	16,238	18,295	685	77.11	4.99	*Enterococcus* phage AUEF3	tail protein	0 (93%)	1 TMD
19/+	18,304	18,597	97	11.25	3.96	*Enterococcus* phage EFAP-1	hypothetical protein	1,00E-53 (85%)	-
20/-	18,776	19,021	81	9.13	5.70	*Enterococcus* phage EFAP-1	hemolysin XhlA family protein	4,00E-50 (100%)	1 TMD
21/-	19,036	19,272	78	8.61	10.04	*Enterococcus* phage EfaCPT1	holin	9,00E-48 (100%)	2 TMD; 1SP PF04531 (Phage_holin_1)
22/-	19,269	20,255	328	36.69	6.52	*Enterococcus* phage EfaCPT1	lysin, N-acetylmuramoyl-L-alanine amidase	0 (98%)	PF01510.24 (Amidase_2)
23/-	20,336	20,563	75	8.84	4.23	*Enterococcus* phage EfaCPT1	glutaredoxin	1,00E-42 (88%)	-
24/-	20,560	21,159	199	23.01	9.53	*Synechococcus* phage S-SSM7	hypothetical protein	9,00E-80 (61%)	-
25/-	21,222	23,513	763	87.50	5.04	*Enterococcus* phage vB_EfaS_IME196	DNA polymerase	0 (98%)	-
26/-	23,548	24,279	243	28.27	6.91	*Enterococcus* phage SANTOR1	DNA methylase	1,00E-162 (98%)	1 TMD

27/-	24,314	24,538	74	8.32	9.98	*Enterococcus* phage EfaCPT1	hypothetical protein	1,00E-43 (95%)	-
28/-	24,609	25,316	235	26.49	4.33	*Enterococcus* phage EfaCPT1	hypothetical protein	6,00E-166 (97%)	-
29/-	25,395	25,646	83	9.59	4.48	*Enterococcus* phage vB_EfaS_IME196	hypothetical protein	3,00E-51 (99%)	-
30/-	25,647	25,937	96	11.31	4.63	*Enterococcus* phage AUEF3	hypothetical protein	6,00E-48 (80%)	-
31/-	25,938	26,756	272	31.99	4.80	*Enterococcus* phage AUEF3	DUF1351 protein	0 (99%)	-
32/-	26,746	26,934	62	7.50	5.05	*Enterococcus* phage IME-EF4	hypothetical protein	7,00E-37 (100%)	-
33/-	26,910	27,686	258	30.05	5.56	*Enterococcus* phage IME_EF3	beta-lactamase superfamily domain protein	3,00E-180 (94%)	-
34/-	27,697	28,176	159	18.68	8.18	*Enterococcus* phage IME_EF3	HNH homing endonuclease-like protein	2,00E-114 (99%)	-
35/-	28,173	28,391	72	8.54	4.23	*Enterococcus* phage IME_EF3	hypothetical protein	1,00E-43 (99%)	-
36/-	28,393	28,974	193	22.57	7.75	*Enterococcus* phage IME_EF3	DUF3310 protein	7,00E-112 (84%)	-
37/-	29,134	29,340	68	8.00	9.51	*Enterococcus* phage IME-EF4	hypothetical protein	1,00E-43 (100%)	-
38/-	29,333	30,076	247	28.00	5.45	*Enterococcus* phage EfaCPT1	prim-pol domain protein	0 (99%)	-
39/-	30,088	30,276	62	7.07	3.86	*Enterococcus* phage IME-EF4	hypothetical protein	2,00E-36 (100%)	-
40/-	30,289	30,486	65	7.83	4.89	*Enterococcus* phage EfaCPT1	hypothetical protein	2,00E-40 (100%)	1 TMD
41/-	30,546	30,722	58	6.86	9.89	*Enterococcus* phage EfaCPT1	hypothetical protein	1,00E-32 (100%)	-
42/-	30,719	32,014	431	49.61	9.94	*Enterococcus* phage EfaCPT1	helicase	0 (94%)	-
43/-	32,007	32,393	128	14.83	4.10	*Enterococcus* phage IME-EF4	endonuclease	3,00E-81 (95%)	-
44/-	32,393	32,599	68	7.58	3.78	*Enterococcus* phage IME-EF4	hypothetical protein	6,00E-40 (100%)	-
45/-	32,601	32,762	53	5.75	3.61	*Enterococcus* phage Ec-ZZ2	hypothetical protein	5,00E-28 (100%)	2 TMD
46/-	32,764	33,048	94	10.52	4.66	*Enterococcus* phage IME-EF4	hypothetical protein	2,00E-52 (90%)	-
47/-	33,184	33,645	153	17.68	10.54	*Enterococcus* phage Ec-ZZ2	hypothetical protein	3,00E-108 (99%)	-
48/-	33,719	33,907	62	7.41	5.12	*Enterococcus* phage EfaCPT1	hypothetical protein	3,00E-35 (100%)	-
49/-	33,996	35,576	526	60.35	5.41	*Enterococcus* phage vB_EfaS_IME196	DNA primase/helicase	0 (99%)	-
50/-	35,671	35,868	65	7.09	9.70	*Enterococcus* phage EfaCPT1	hypothetical protein	8,00E-24 (89%)	2 TMD
51/-	35,865	36,104	79	9.69	4.80	*Enterococcus* phage IME_EF3	hypothetical protein	3,00E-48 (96%)	-
52/-	36,101	36,262	53	6.41	4.64	*Enterococcus faecalis*	hypothetical protein	3,00E-24 (94%)	-
53/-	36,259	36,477	72	8.33	6.24	*Enterococcus* phage EfaCPT1	hypothetical protein	5,00E-42 (93%)	-
54/-	36,477	36,698	73	8.54	3.73	*Enterococcus* phage vB_EfaS_IME196	hypothetical protein	4,00E-41 (95%)	-
55/-	36,710	36,892	60	7.11	3.87	*Enterococcus* phage AUEF3	hypothetical protein	3,00E-36 (100%)	-
56/-	37,048	37,443	131	15.42	4.74	*Enterococcus* phage AUEF3	hypothetical protein	2,00E-67 (80%)	-
57/-	37,444	37,761	105	13.02	4.52	*Enterococcus* phage IME-EF4	hypothetical protein	1,00E-56 (87%)	-

58/-	37,850	38,059	69	7.97	4.21	*Enterococcus* phage AUEF3	hypothetical protein	1,00E-31 (82%)	-
59/-	38,056	38,241	61	7.12	4.28	*Enterococcus* phage SANTOR1	hypothetical protein	3,00E-33 (95%)	-
60/-	38,241	38,648	135	15.74	5.01	*Enterococcus* phage AUEF3	hypothetical protein	1,00E-94 (99%)	-
61/+	38,645	38,857	70	8.19	5.07	*Enterococcus* phage vB_EfaS_IME196	hypothetical protein	8,00E-32 (84%)	-
62/+	38,895	39,260	121	14.72	4.74	*Enterococcus* phage SANTOR1	hypothetical protein	1,00E-42 (71%)	-
63/+	39,273	39,590	105	12.59	9.69	*Enterococcus* phage vB_EfaS_IME196	hypothetical protein	3,00E-64 (90%)	-
64/+	40,094	40,297	67	7.56	9.83	*Enterococcus* phage EfaCPT1	hypothetical protein	2,00E-38 (99%)	2 TMD
65/+	40,380	40,586	68	7.81	5.60	*Enterococcus* phage EfaCPT1	hypothetical protein	2,00E-42 (100%)	-

Table S2. Genomic features of the *Enterococcus faecium* phage Zip.

			Predicted protein						
**ORF (+/-)**	**Start (bp)**	**Stop (bp)**	**Size (aa)**	**MW (kDa)**	**pI**	**Best species hit**	**Putative function**	**E-value (% identify)**	**Signals/Motifs**
1/+	191	535	114	12.68	3.38	*Enterococcus* phage vB_EfaP_IME199	hypothetical protein	4,00E-27 (74%)	-
2/+	550	714	54	6.02	3.99	*Enterococcus* phage vB_EfaP_IME199	hypothetical protein	2,00E-25 (87%)	-
3/+	716	1,888	390	44.26	5.85	*Enterococcus* phage vB_EfaP_IME199	major capsid	0.0 (95%)	-
4/+	1,908	2,933	341	38.59	5.15	*Enterococcus* phage vB_EfaP_IME199	phage connector	0.0 (94%)	-
5/+	2,890	3,543	217	25.29	5.15	*Enterococcus* phage vB_EfaP_IME199	lower collar protein	1,00E-155 (98%)	-
6/+	3,556	4,980	474	52.84	4.69	*Enterococcus* phage vB_EfaP_IME199	metallophosphatase	1,00E-155 (98%)	-
7/+	4,991	5,785	264	29.91	7.16	*Enterococcus* phage vB_EfaP_IME199	hypothetical protein	0.0 (97%)	-
8/+	5,787	7,181	464	52.17	4.41	*Enterococcus* phage vB_EfaP_IME199	hypothetical protein	0.0 (75%)	-
9/+	7,193	8,944	583	66.04	6.96	*Enterococcus* phage vB_EfaP_IME199	tail fiber	0.0 (98%)	-
10/+	8,989	9,210	73	8.75	4.32	*Enterococcus* phage vB_EfaP_IME199	holin	3,00E-39 (89%)	1 TMD PF10960.7 (Holin_BhlA)
11/+	9,207	10,121	304	33.08	10.14	*Enterococcus* phage vB_EfaP_IME199	endolysin	0.0 (93%)	PF01510.24 (Amidase_2)
12/-	10,185	12,194	669	72.29	6.43	*Enterococcus* phage vB_EfaP_IME199	CHAP-containing domain	0.0 (84%)	PF05257.15 (CHAP)
13/-	12,197	12,751	184	21.74	9.94	*Enterococcus* phage vB_EfaP_IME199	HNH endonuclease	9,00E-115 (85%)	-
14/-	12,748	12,978	76	9.08	9.84	*Enterococcus* phage vB_EfaP_IME199	hypothetical protein	2,00E-44 (96%)	-
15/-	12,983	13,135	50	5.86	4.08	*Enterococcus* phage vB_EfaP_IME199	hypothetical protein	2,00E-20 (81%)	-
16/-	13,132	13,320	62	7.47	8.88	*Enterococcus* phage vB_EfaP_IME199	hypothetical protein	2,00E-32 (85%)	-
17/-	13,398	15,737	779	91.38	5.34	*Enterococcus* phage vB_EfaP_IME199	DNA polymerase	0.0 (95%)	-
18/-	15,77	17,008	412	48.80	6.07	*Enterococcus* phage vB_EfaP_IME199	encapsidation protein	0.0 (96%)	-
19/-	17,022	17,480	152	17.72	10.00	*Enterococcus* phage vB_EfaP_IME199	hypothetical protein	8,00E-106 (98%)	-
20/-	17,483	17,905	140	15.84	3.87	*Enterococcus* phage vB_EfaP_IME199	hypothetical protein	1,00E-67 (86%)	1 TMD
21/-	17,982	18,311	109	12.14	10.10	*Enterococcus* phage vB_EfaP_IME199	single-stranded DNA-binding protein	2,00E-64 (93%)	-
22/-	18,388	18,615	75	9.24	5.65	-	-	-	
